# Supplementary material for: Anaerobic microsites have an unaccounted role in soil carbon stabilization
Source: Nat Commun. 2017 Nov 24;8:1771. doi: 10.1038/s41467-017-01406-6 (PMC5701132; doi:10.1038/s41467-017-01406-6)
Supplement: Supplementary file 1 — Supplementary Information [file 41467_2017_1406_MOESM1_ESM.pdf]

**Supplementary Table 1:** Basic soil characteristics of upland soils differing in particle size (or texture) used in this study

| **Soil Classification** | **Depth** | **Horizon** | **Bulk density** | **Clay content** | **CEC$_{eff}$** | **pH** | **Total C** | **C/N** | **WEOC** | **POC** | **MAOC** |
|---|---|---|---|---|---|---|---|---|---|---|---|
| | cm | | g cm$^{-3}$ | % | mmol$_c$ kg$^{-1}$ | | % | | | % of total C | |
| *a) Coarser-textured soil profile:* | | | | | | | | | | | |
| Fine silty, mixed, superactive mesic Ultic Argixeroll | 20 | $A_h$ | 1.26 | 27.6 | 321 | 5.1 | 1.9 | 10.6 | 7.5 | 4.0 | 96.0 |
| | 100 | $B_t$ | 1.31 | 28.4 | 353 | 5.9 | 0.22 | 3.7 | 0.8 | 3.2 | 96.8 |
| *b) Finer-textured soil profile:* | | | | | | | | | | | |
| Fine loamy, mixed, superactive, mesic Argiaquic Xeric Argialboll | 20 | $A_h$ | 1.40 | 20.3 | 293 | 5.2 | 1.7 | 9.5 | 9.8 | 3.3 | 96.7 |
| | 100 | $B_t$ | 1.40 | 32.2 | 389 | 6.2 | 0.24 | 3.00 | 1.4 | 4.3 | 95.7 |

**Supplementary Table 2:** Summary of steps in carbon oxidation rate calculations for different respiration pathways

| Rate | Measurements and calculations |
|---|---|
| Total respiration rate (TotalR) | Measured $CO_2$ production rate in effluent |
| Aerobic respiration (AerobicR) | Calculated TotalR – AnaerobicR |
| Anaerobic respiration (AnaerobicR) | Measured $CO_2$ production rate with depth in anaerobic incubations |
| Denitrification (Denitr.) | Measured denitrification rate using $^{15}N\text{-}NO_3^-$ |
| Mn reduction (MnR) | Measured decrease in reducible Mn relative to $t_o$ |
| Fe reduction (FeR) | Measured decrease in reducible Fe relative to $t_o$ |
| Methanogenesis (Meth.) | Measured $CH_4$ production rate with depth in anaerobic incubations |

**Supplementary Table 3:** Stoichiometry of organic carbon oxidation pathways

| | |
|---|---|
| Aerobic respiration | $CH_2O + O_2 \rightarrow H_2O + CO_2$ |
| Denitrification | $4/5\ H^+ + CH_2O + 4/5NO_3^- \rightarrow CO_2 + 2/5N_2 + 7/5H_2O$ |
| Mn reduction | $4H^+ + CH_2O + 2MnO_2 \rightarrow 2Mn^{2+} + 2H_2O + CO_2$ |
| Fe reduction | $8H^+ + CH_2O + 4FeOOH \rightarrow 4Fe^{2+} + CO_2 + 7H_2O$ |
| Sulfate reduction | $H^+ + CH_2O + 1/2SO_4^{2-} \rightarrow CO_2 + 1/2H_2S + H_2O$ |
| Methanogenesis | $2CH_2O \rightarrow CH_4 + CO_2$ |

**Supplementary Table 4:** Fitting parameters for peak deconvolution of NEXAFS spectra

| **Functional group assignments** | **Energy (eV)** | **Full width at half maximum** |
|---|---|---|
| quinonic or polycondensed | 284.05 | 0.5 |
| aromatic C | 285.05 | 0.5 |
| phenolic C | 286.2 | 0.5 |
| aliphatic C | 287.25 | 0.5 |
| carboxylic C | 288.35 | 0.5 |
| alkyl-OH | 289.35 | 0.5 |
| carbonyl and carbonate | 290.3 | 0.5 |
| sigma* | 292.0 | 3.0 |
| sigma* | 300.0 | 3.0 |
| arctangent | 290.0 | 0.5 (height = 1.0) |

**Supplementary Table 5:** Peak deconvolution for C NEXAFS spectra of top- (20 cm) and subsoil (100 cm) horizons of coarse- and fine-textured upland soils

| **Carbon functional group** | **Coarser texture 20 cm** | **Finer texture 20 cm** | **Coarser texture 100 cm** | **Finer texture 100 cm** |
|---|---|---|---|---|
| Quinonic C | 4.4 | 4.4 | 0.0 | 2.0 |
| Aromatic C | 13.1 | 13.6 | 12.7 | 15.6 |
| Phenolic C | 9.6 | 8.5 | 1.4 | 0.9 |
| Aliphatic C | 8.4 | 8.0 | 11.7 | 12.5 |
| Carboxylic/Amide C | 36.0 | 36.5 | 43.6 | 38.0 |
| Alkyl C | 24.2 | 23.6 | 27.1 | 24.7 |
| Carbonyl C=O | 4.2 | 5.3 | 3.7 | 6.3 |

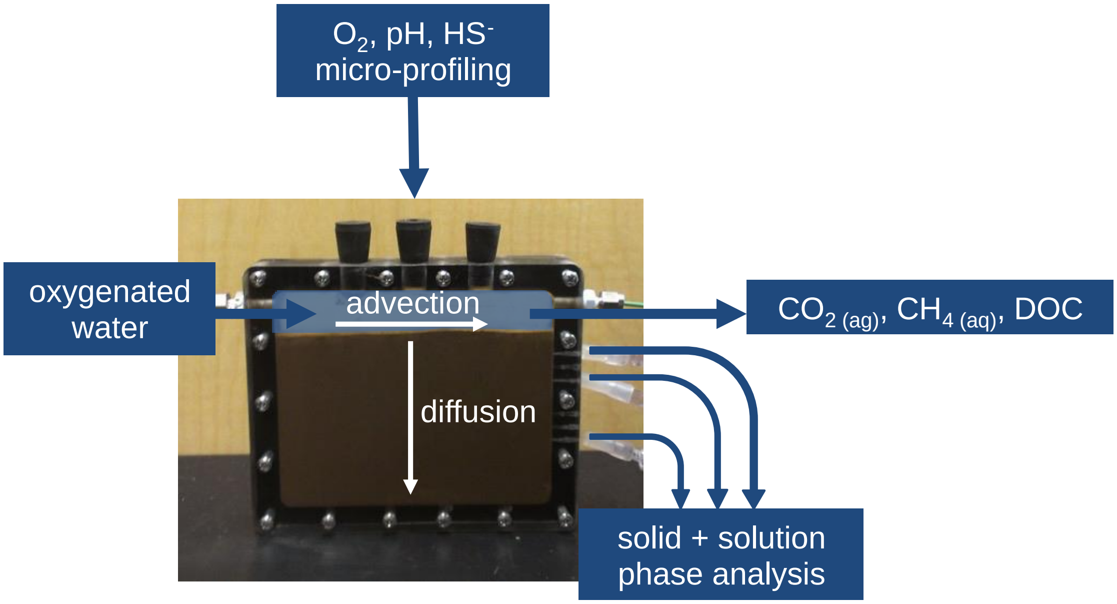


**Supplementary Fig. 1:** Diffusion-limited flow reactor. The flow channel (advective domain) contained oxygenated nutrient solution supplied at a constant rate using a peristaltic pump. Mixtures of natural soil with different quartz grain sizes were used to vary soil texture (within the diffusion-limited domain).

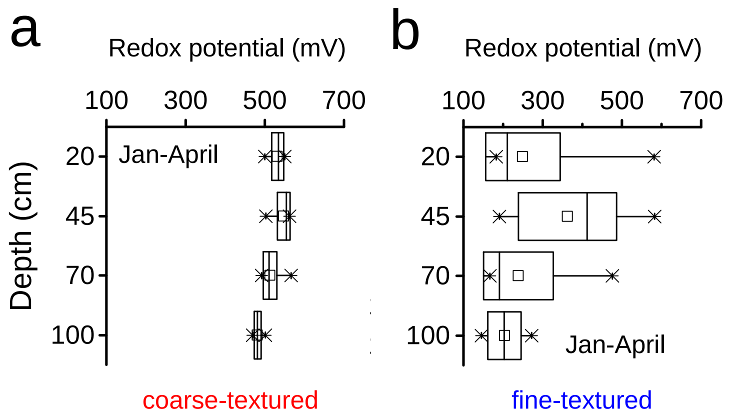


**Supplementary Fig. 2:** Range of redox potential ($E_h$) for coarse- and fine-textured soil profiles during the wet season (Jan-April), showing mean (dot), median (line), one standard deviation (box), and 5 to 95% intervals (whiskers). Data was obtained using 5 replicate platinum redox probes permanently installed at 10, 20, 50, and 100 cm depth in both profiles. A custom amplifier was used to mitigate signal drift on the handheld digital multimeter.

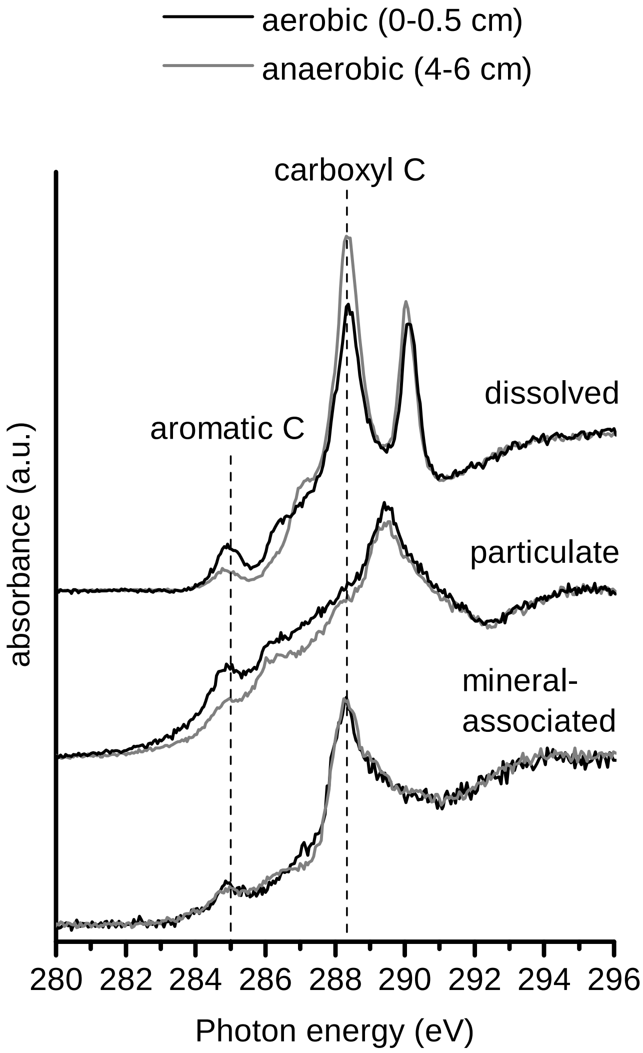


**Supplementary Fig. 3:** C 1s NEXAFS spectra of dissolved, particulate, and mineral-associated organic carbon pools in the anaerobic and anaerobic zones isolated by density fractionation. Spectra were only collected for the fine texture treatment. Dashed lines indicate the 1s–p* transition of C in aromatic C=C bonds and the 1s–p* transition of C in carboxylic groups at 288.3 eV. The relative absorbance of carboxylic and aromatic C was quantified by peak deconvolution and used as a proxy for oxidation state in this study (see Fig. 3 and 4 in main manuscript text).

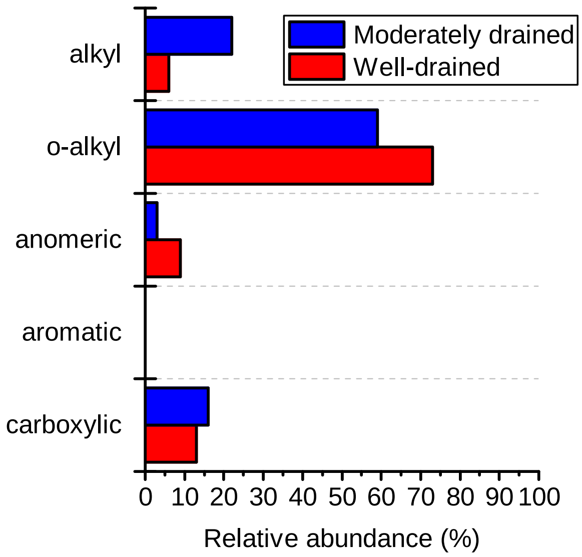


**Supplementary Fig. 4:** Comparison of C functional group abundance in water extracts of coarser- and finer-textured upland soils based solution-state $^{13}C$ NMR spectra. Relative abundances were obtained using a peak integration of $^{13}C$ NMR spectra as described in the text. The relative abundance of alkyl C in the fine-textured subsoil (100 cm-depth) is three times greater than the amount present in coarse-textured subsoil. Alkyl C in $^{13}C$ NMR spectra is generally attributed to aliphatic compounds such as waxes and fatty acids, suggesting the preferential preservation of these water-extractable (and thus potentially bioavailable), reduced compounds in finer-textured upland soils.

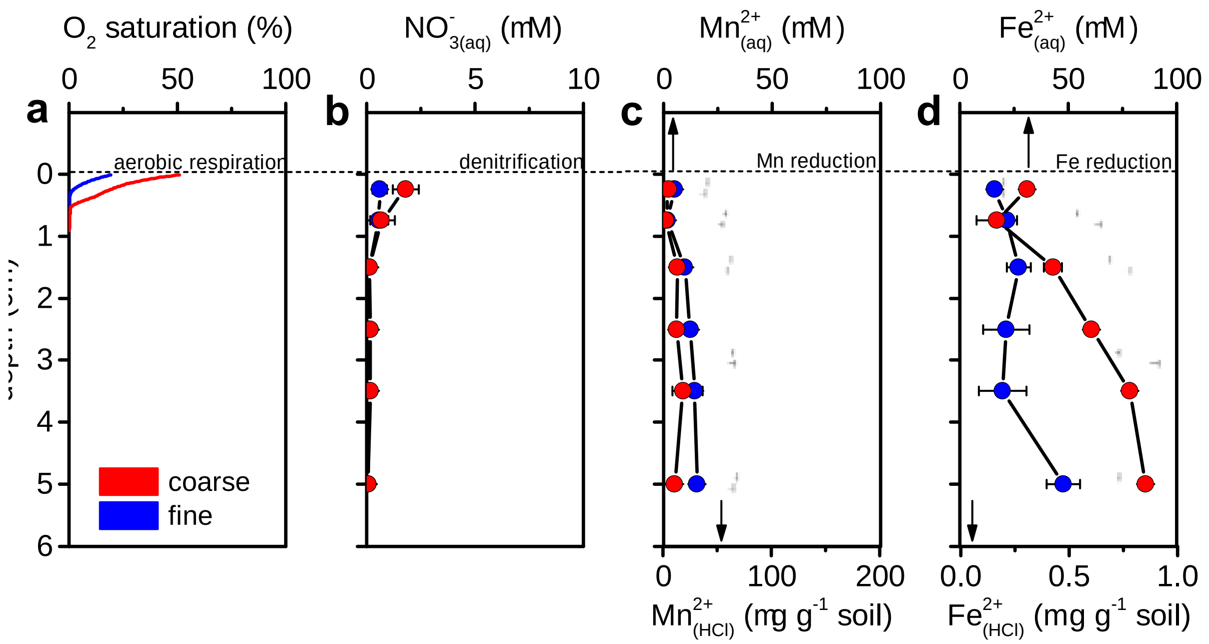


**Supplementary Fig. 5:** Changes in the distribution of oxygen and alternative terminal electron acceptors across the diffusion-limited domain as a function of soil texture. Oxygen profiles were recorded at day 35 of the experiment and represent the average of two measurements for each reactor. Subscripts (aq) and (HCl) denote concentrations in pore water or HCl extracts, respectively.

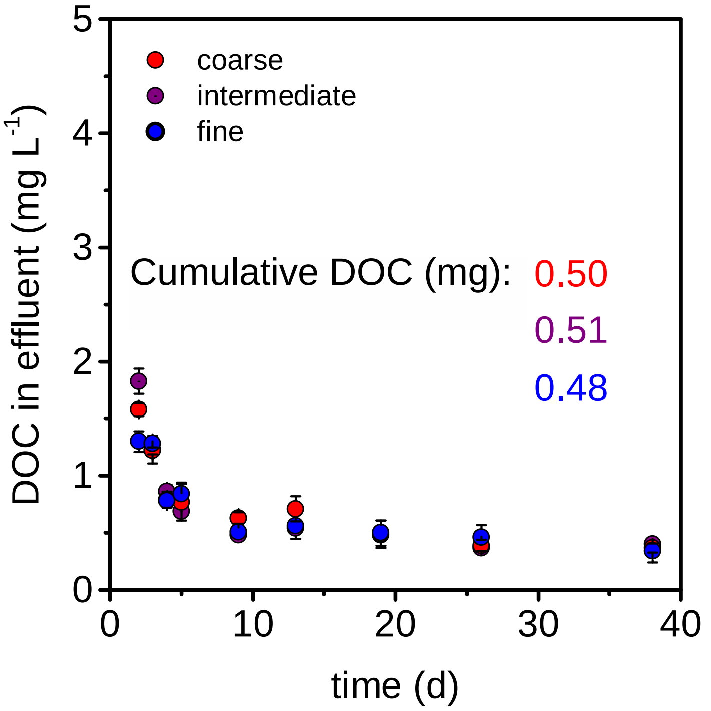


**Supplementary Fig. 6:** Dissolved organic carbon (DOC) concentrations in reactor effluent. The inset indicates the total cumulative loss of DOC from the reactor over the experimental period. Error bars denote the standard error of the mean of four replicate reactors.

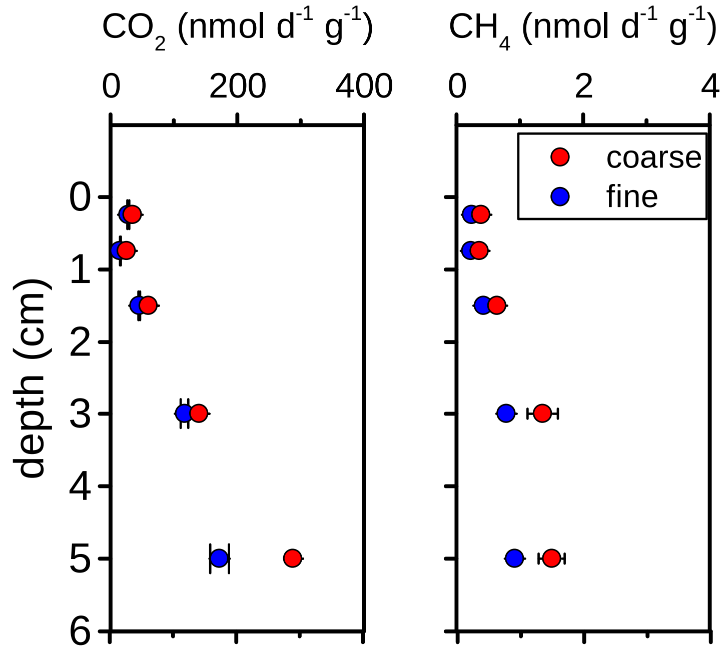


**Supplementary Fig. 7:** $CO_2$ and $CH_4$ productions rates with depth as a function of depth of texture. Rates were determined in anaerobic incubations of soil material samples from each depth after harvest of the reactors.

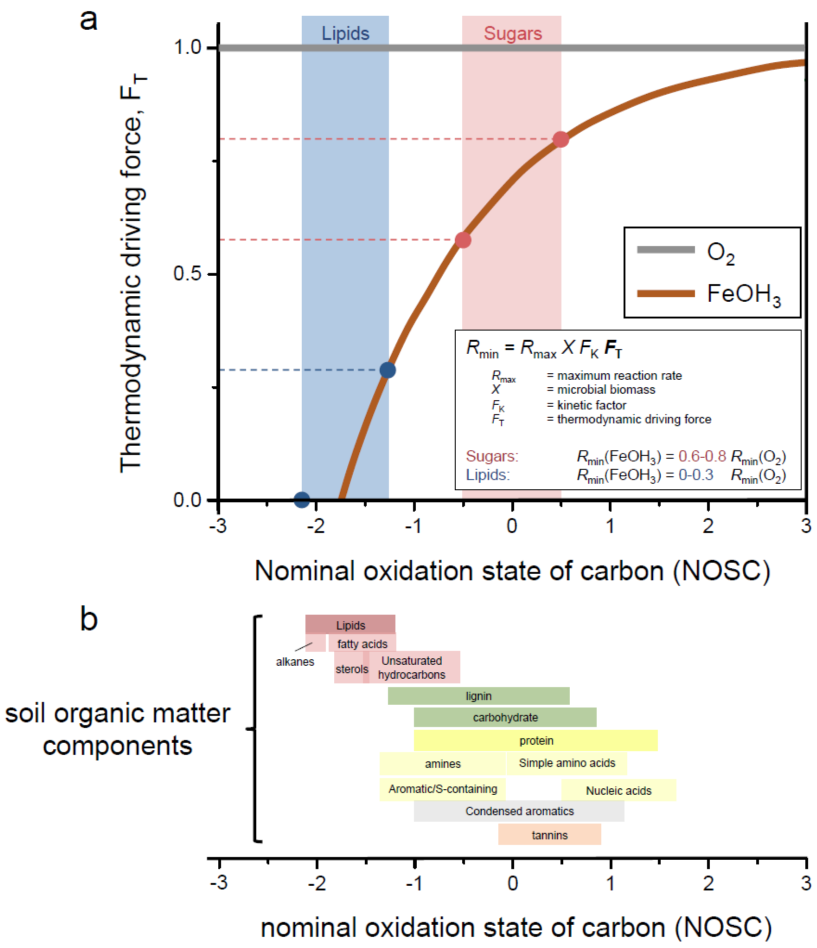


**Supplementary Fig. 8:** Thermodynamic constraints on the oxidation of organic compounds under anaerobic conditions. a) Thermodynamic driving force, $F_T$, for the oxidation of organic compounds as a function of their nominal oxidation state of carbon (NOSC). b) Range of NOSC values for compound classes commonly present in soil organic matter. NOSC values are estimated based on the approximate position of each compound class in Van Krevelen diagrams. When coupled to oxygen, $F_T$ for the oxidation of an organic compound is close to 1. The reaction thus is expected to proceed almost uninhibited for compounds spanning the full range of NOSCs. Consequently, the mineralization (R*min*, equation 1 in main text) would be the same for reduced compounds with low NOSC (e.g., lipids) and more oxidized compounds with high NOSC (e.g., sugars). However, when oxidation an organic compounds is coupled to the reduction of Fe(III), the most abundant terminal electron acceptor in upland soil systems, a very different scenario results. $F_T$ for sugars with NOSCs of around zero decreases to 0.70, reducing $R_{min}$ by 30% relative to aerobic conditions. or reduced substrates such as lipids, this decrease is even more dramatic. $F_T$ for lipids with NOSCs of less than -1.7 is zero, and microbial oxidation is thermodynamically inhibited. As a consequence, $R_{min}$ will approach zero for compound classes such as lipids and fatty acids under anaerobic conditions. Even for less oxidized lipids and fatty acids ($F_T$ ~-1.3), $R_{min}$ would be reduced by 70% relative to aerobic rates. It follows that under Fe reducing conditions, $R_{min}$ is expected to be substantially higher for oxidized compounds such as sugars than for more reduced compounds such as lipids and fatty acids. For further detail, the reader is referred to the *Thermodynamic Constraints on Carbon Oxidation* section in the Methods section.

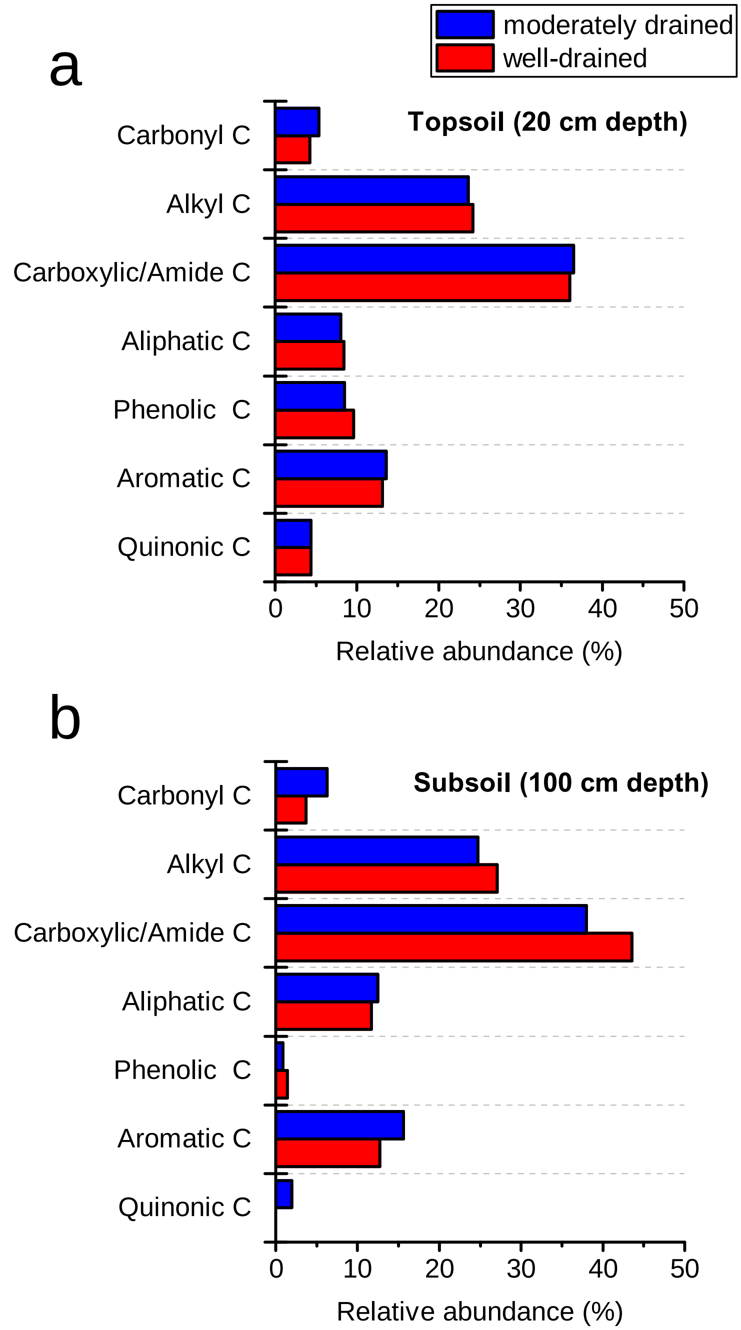


**Supplementary Fig. 9:** Comparison of C functional group abundance in coarser- and finer-textured upland soils using C NEXAFS. Relative abundances were obtained using a peak deconvolution of C NEXAFS spectra described in the text. The topsoils (20 cm-depth) show only minor differences. However, the contribution of reduced functional groups (e.g., aromatic and aliphatic C) is significantly greater in finer-textured subsoil than in coarser textured subsoil horizons (100 cm-depth). Conversely, the abundance of more oxidized functional groups (e.g., carboxylic and alkyl C) decreases in fine-textured subsoil horizons.
